# Similar Microbial Communities Found on Two Distant Seafloor Basalts

**DOI:** 10.3389/fmicb.2015.01409

**Published:** 2015-12-16

**Authors:** Esther Singer, Lauren S. Chong, John F. Heidelberg, Katrina J. Edwards

**Affiliations:** ^1^Joint Genome Institute, Walnut CreekCA, USA; ^2^Department of Earth Sciences, University of Southern California, Los AngelesCA, USA; ^3^Department of Marine Environmental Biology, University of Southern California, Los AngelesCA, USA

**Keywords:** seafloor basalt, metagenome, thaumarchaeota, microbe-rock interactions, oceanic crust

## Abstract

The oceanic crust forms two thirds of the Earth’s surface and hosts a large phylogenetic and functional diversity of microorganisms. While advances have been made in the sedimentary realm, our understanding of the igneous rock portion as a microbial habitat has remained limited. We present the first comparative metagenomic microbial community analysis from ocean floor basalt environments at the Lō’ihi Seamount, Hawai’i, and the East Pacific Rise (EPR; 9°N). Phylogenetic analysis indicates the presence of a total of 43 bacterial and archaeal mono-phyletic groups, dominated by *Alpha-* and *Gammaproteobacteria*, as well as *Thaumarchaeota*. Functional gene analysis suggests that these *Thaumarchaeota* play an important role in ammonium oxidation on seafloor basalts. In addition to ammonium oxidation, the seafloor basalt habitat reveals a wide spectrum of other metabolic potentials, including CO_2_ fixation, denitrification, dissimilatory sulfate reduction, and sulfur oxidation. Basalt communities from Lō’ihi and the EPR show considerable metabolic and phylogenetic overlap down to the genus level despite geographic distance and slightly different seafloor basalt mineralogy.

## Introduction

Oceanic basalts cover approximately 60% of the Earth’s surface. Due to their high permeability, these volcanic rocks are greatly influenced by infiltration and circulation of seawater (Fisher, 1998; Fisher and Becker, 2000). The rock-seawater interaction results in a flux of energy and solutes between basalt crust and the overlying seawater (Fisher, 1998). Micro-niches on and inside basalts support both autotrophic and heterotrophic microbial growth. Lava surfaces are predominantly composed of volcanic glass, harboring reduced elemental species, including iron, sulfur, and manganese (Alt, 1995). These constituents can be oxidized by chemoautotrophic microorganisms with oxygen and nitrate to fix CO_2_ (Edwards et al., 2003b). Furthermore, ferromanganese crust formation on the glassy rims of pillow basalts can give rise to the formation of microbial biofilms, which use these secondary minerals for energy metabolism (Templeton et al., 2009).

Cell densities in volcanic glasses from the East Pacific Rise (EPR) were estimated to amount to 10^5^–10^9^ cells g^-1^ basalt based on qPCR results (Einen et al., 2008; Santelli et al., 2008). Cell densities and community compositions are hypothesized to be linked to the alteration state of the source rock: younger basalts generally host less diverse communities that are more similar to their source environments (e.g., surrounding water), whereas older basalts (>20,000 years) usually display richer communities adapted to the mineralogical composition of the rock (Santelli et al., 2008). Fe(II)-reducing bacteria alone were estimated to amount to 10^3^ cells ml^-1^ at the Lō’ihi Seamount (Emerson, 2009). While the origin of these seafloor basalt microbial communities may be the surrounding seawater and/or sediments, over time (thousands of years), they appear to become distinct from the surrounding habitat, so that mineralogy – rather than geographical setting – ultimately determines the community structure (Santelli et al., 2008; Toner et al., 2013). While deep-sea basalts host a greater phylogenetic diversity of bacteria and archaea than other deep-sea environments, community structures on basalts of similar mineralogical composition resemble each other when compared to other environments (Huber et al., 2003; Santelli et al., 2008). In fact, shared operational taxonomic unit (OTU) richness and community membership between the EPR and Hawai’i suggest that there is a basalt biome which positively correlates with seafloor basalt characteristics (Santelli et al., 2009). Microbial community richness is most likely supported by the wide variety of micro-niches that allows diverse redox reactions and metabolic pathways (e.g., heterotrophic, anaerobic, and reductive) within small spatial scales (Santelli et al., 2008; Shi et al., 2012).

Thus far, the limited number of existing molecular microbial community studies on seafloor basalts exposed to seawater have all relied on PCR-biased results. Thorseth et al. (2001) investigated samples from Knipovich Ridge and observed bacteria belonging to the *Proteobacteria, Bacteroidetes*, and archaea. Lysnes et al. (2004) studied Hawaiian basalts and determined that the microbial community was dominated by bacteria and was structurally dependent on rock age. Santelli et al. (2008) found 10^5^–10^6^ cells almost exclusively from the bacterial domain in the glassy rind of seafloor basalts from the Norwegian/Greenland sea and did not find a correlation between community structure and rock age. The large diversity and richness of microbial communities on seafloor basalts from various locations was first emphasized in studies from Mason et al. (2007, 2009), Santelli et al. (2008), and microbial community structures appeared comparable between geographically distant sites. Santelli et al. (2008) and Mason et al. (2009) found similar relative abundances of *Proteobacteria* (56–68%), *Planctomycetes* (5–8%), *Actinobacteria* (7–9%), *Bacteroidetes* (1–10%), *Acidobacteria* (3–6%) at Lō’ihi, the EPR, and the Juan de Fuca Ridge. Archaea were found to be ubiquitous in seafloor basalts, although estimates of archaeal relative abundance are discrepant, ranging from 0.02 to 25% (Einen et al., 2008; Santelli et al., 2008; Mason et al., 2009).

Since metabolic functions are difficult to construe from 16S rRNA phylogeny, it is unclear how functional gene patterns compare among rocks from geographically different locations. Functional surveys conducted at the Lō’ihi Seamount and the EPR, to date, include isolate studies (Templeton et al., 2005; Emerson, 2009), synchrotron-based X-ray microprobe mapping (Templeton et al., 2009), enzyme assays (Jacobson Meyers et al., 2014), and stable isotope incubations of crustal samples (Orcutt et al., 2015). These studies have helped characterize the extent of some of the main microbial metabolic activities supported at these sites. While these studies elegantly investigated specific metabolic pathways, studies of the pathway diversity present at seafloor basalts are scarce. Mason et al. (2009) have provided the only broad functional gene analysis at the EPR and the Juan de Fuca Ridge using GeoChip. The authors detected genes for carbon fixation, methane oxidation, methanogenesis, and nitrogen fixation on rocks from the EPR and the Juan de Fuca Ridge (Mason et al., 2009). In order to further test the hypothesis (while avoiding PCR-bias) that the phylogeny and functional gene set of microbial communities is comparable between seafloor basalts of similar age and mineralogy, we conducted the first comprehensive metagenomic study of seafloor basalts. We describe the phylogenetic and metabolic characteristics of the basalt-hosted microbial communities from the Lō’ihi Seamount and the EPR, and provide links to the mineralogy of the seafloor basalts and parameters of the surrounding environment.

## Materials and Methods

### Rock Collection and Analysis

**FIGURE 1:**
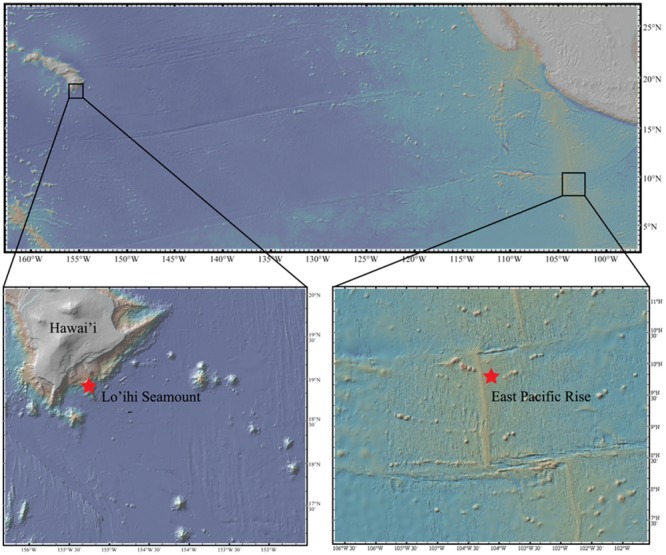
**Map of study sites**.

One sample of seafloor basalt (AT11-07_3968_B_OF5) was collected from the EPR (9° 43.8′ N, 104° 9.6′ W) from a depth of 2,674 m aboard the R/V Atlantis using the submarine Alvin (cruise AT11-07) in 2004. Two seafloor basalt samples (J2-243 R2-F, J-246 R2) were collected from the Lō’ihi Seamount (18° 28.2′ N, 155° 10.8′ W) at a depth of 5,000 m aboard the R/V Melville using the ROV Jason II in 2006 (**Figure 1**). All seafloor basalts were stored frozen at -80°C for XRD analysis and DNA extraction. Bulk mineralogy analysis, i.e., quantitative determination of rock-forming minerals and total clay minerals, was determined on all three seafloor basalts via X-ray Diffraction (XRD) analysis at KT GeoServices, Inc. Detection limits were at 1–5 wt%. The two Lō’ihi seafloor basalts were combined for analysis.

### DNA Extraction and Sequencing

DNA was extracted from basalt chips using a phenol-chloroform extraction with a negative control (NC). DNA extracts from the two Lō’ihi seafloor basalt samples were combined. Since the amount of DNA was <1 μg on all seafloor basalts, DNA was amplified using the illustra GenomiPhi V2 DNA Multiple Displacement Amplification (MDA) kit (GE Healthcare Life Sciences, Pittsburgh, PA, USA). The NC sample was also processed with the MDA kit in the same reaction as the seafloor basalt samples. Final DNA samples and the control were sent to the core genomics center at the University of Pennsylvania for whole genome shotgun sequencing on a Roche GS-FLX Titanium 454 sequencer (454 Life Sciences, Branford, CT, USA).

### Sequence Processing and Assembly

Raw sequence reads were evaluated with FastQC version 0.11.3 (Schmieder and Edwards, 2011a), quality trimmed (minimum quality score–25, maximum length–450 bp, maximum homopolymer length–9 bp, max N-tail–1 bp), and filtered (removal of technical duplicates, minimum length–60 bp) with Prinseq 0.20.4 (Schmieder and Edwards, 2011b) and MG-RAST (Meyer et al., 2008). We obtained 1,102,191 sequences in the Lō’ihi dataset, 1,191,651 sequences in the EPR dataset, and 58,188 sequences in the NC dataset. Quality-filtered reads were assembled *de novo* using standard 454 settings in mira 3.4.1.1 (Chevreux et al., 1999). Padded (i.e., including potential gaps) contigs >500 bp were filtered using mira 3.4.1.1 (convert_project; Chevreux et al., 1999).

Seafloor basalt contigs were screened for contamination using a combination of BBMap (bbduk.sh with parameters *mcf* = 0.25, *k* = 31) and the BLASTN algorithm (Altschul et al., 1990). The BBMap algorithm identified 10 potentially contaminant contigs in the Lō’ihi (total of 12,423 bp) and four potentially contaminant contigs in the EPR metagenome dataset (total of 4,290 bp). For the Lō’ihi dataset, the BLAST alignment results for the identified contaminant contigs rendered ≥84% identity over ≥28% of the seafloor basalt contig length. For the EPR dataset, the BLAST alignment results for the contaminant contigs rendered ≥86% identity over ≥8% of the seafloor basalt contig length. Contig statistics are displayed in **Table 1** and in **Supplementary Figure S1**. All sequences are available online at http://www.bco-dmo.org/dataset/616326.

**Table 1:** Sequence, assembly, and annotation statistics of metagenome datasets.

Sequences: post QC											
Sample	Depth (m)		Total reads		Sequence total (Gbp)		Avg. sequence length (bp)		Average %GC	
Lō’ihi	5,000		1,055,848		∼268		253 ± 110		45	
EPR	2,540		1,191,651		∼233		195 ± 102		48	
NC	N/A		58,188		∼10		177 ± 93		51	

**Contig statistics**											

**Sample**	**# of reads assembled**	**Avg. total coverage**		**# of contigs**		**N50 [bp]**	**Contigs >500 bp**	**BLAST hits^∗^**		**Pfam hits^∗^**

Lō’ihi	245,493	3.94		17,080		877	11,617	9,154		8,668
EPR	89,245	3.43		9,541		763	6,440	5,440		4,377
NC	51,089	6.73		1,080		2638	1,009	1,008		839

*^∗^Filtered BLASTX and Pfam hits as described in section “Materials and Methods.”*

### Gene Annotation

Contigs >500 bp were annotated by using the BLASTX algorithm in diamond v0.79 (Buchfink et al., 2014) against the Refseq non-redundant database (Pruitt et al., 2011) and analyzed in MEGAN5 (Huson and Weber, 2013). Relative abundance comparison of phylogenies between seafloor basalt datasets was achieved by normalizing to total read length (Lō’ihi: 6,738,682 bp, EPR: 3,508,565 bp). Further functional prediction of contigs was performed by alignment against Pfam-A regions from the Pfam version 27.0 database (Finn et al., 2013) using prodigal 2.50 (Markowitz, 2006). Pfam hits with *e*-value <10^-5^ and pfam score >50 were retained. Contig fractions encoding for small subunit (SSU) ribosomal genes were retrieved with RNAmmer-1.2 (Lagesen et al., 2007) and classified using the SINA aligner (Pruesse et al., 2012).

### Phylogenetic Reconstruction and Statistical Analysis

Community richness was estimated using the Chao1 index, and diversity analysis was calculated using the Shannon index in QIIME 1.9.1 (alpha_diversity.py) based on BLASTX assignments of contigs. Phylosift was used to assess community diversity using the core molecular marker set of genes, which includes ∼40 three-domain protein coding genes, single-copy eukaryote specific nuclear orthologs, ribosomal RNA genes (16S/18S), mitochondrial genes (mtDNA markers), and plastid and viral markers identified through Markov-clustering algorithms applied to genome datasets (Darling et al., 2014). *D*-score calculations of functional categories in Supplementary Table S2 were calculated as previously described (Markowitz et al., 2007).

## Results

### Mineralogy

Bulk mineralogy of the basalt from both sites is shown in **Table 2**. Considerable amounts of glass (amorphous), containing ∼10 wt% iron, were identified in both samples. The volcanic glass was mainly composed of silica (≥70%), but also harbored magnesium and iron in the form of Fe_3_O_4_ (magnetite), i.e., Fe^2+^ and Fe^3+^ ions, which is readily oxidized to hematite with oxygen. The Lō’ihi basalt sample additionally contained significant amounts of pyroxene and olivine, which typically consist of ∼6 and 8–34 wt% Fe(II), respectively. Overall, the mineralogies observed were representative of typical basalt compositions (Yoder, 1976).

**Table 2:** X-ray Diffraction analysis data of bulk seafloor mineralogy.

XRD#	Lō’ihi (wt%)	East Pacific Rise (EPR; wt%)
Quartz	1.5	1.7
Pyroxene	37.5	7.1
Olivine	12.5	0
Amorphous	48.5	91.2

### Phylogeny

**FIGURE 2:**
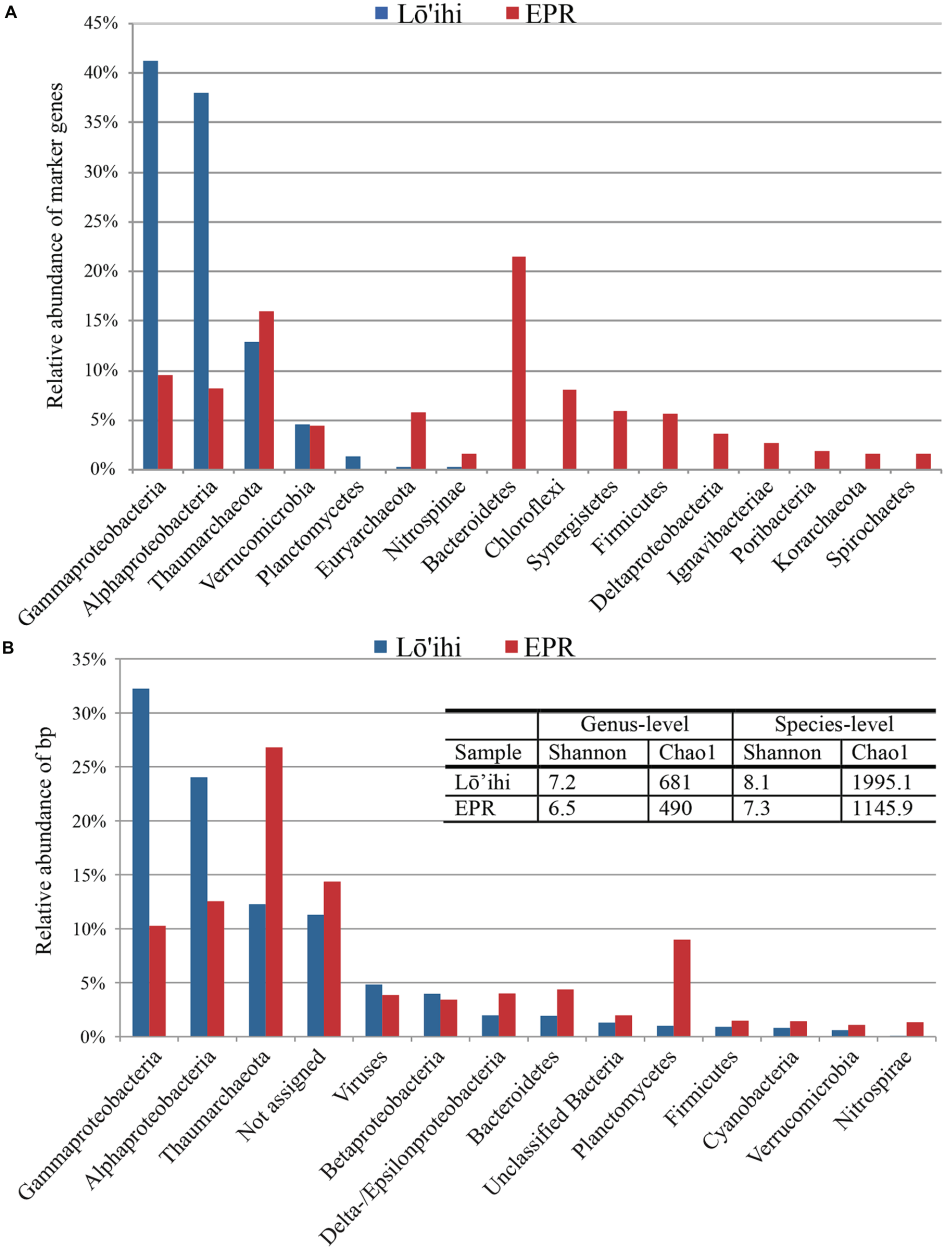
**Phyla classification of taxonomy assignments based on molecular marker genes **(A)** and BLAST assignments (B).** *Proteobacteria* were split into classes due to their inherent diversity. Only phyla/*Proteobacterial* classes with relative abundance >1% are listed. The Lō’ihi community is dominated by few phyla/*Proteobacterial* classes compared to a more even taxa distribution at the EPR **(A)**. Community structure based on BLAST assignment supports the dominance of *Gamma-* and *Alphaproteobacteria* at Lō’ihi, but also displays comparable evenness and diversity to the EPR community. The barplot shows percentages of total identifiable hits to phyla and *Proteobacteria* classes normalized by read length and with relative abundance >1% (Lō’ihi: 6,738,682 bp; EPR: 3,508,565 bp; **B)**.

Phylogenetic analysis using 180 identified molecular marker genes in seafloor basalt metagenomes from Lō’ihi and 125 from the EPR resulted in 13 and 17 monophyletic groups, respectively (**Figure 2A**). At Lō’ihi, *Gamma-* and *Alphaproteobacteria* accounted for nearly 80% of total abundance, with *Thaumarchaeota* (13%) representing the third most abundant phylogenetic group. Besides *Verrucomicrobia* (4.5%) and *Planctomycetes* (1.3%), all other phyla accounted for less than 1% of the total prokaryotic community. The EPR community displayed more evenness; *Bacteroidetes, Thaumarchaeota*, and *Proteobacteria* together held similar shares, comprising over 60% of the total community (**Figure 2A**). Phylogenetic classification of all contigs resulted in 7,841 (67.2% of total) and 4,566 (77.2% of total) contigs assigned to 44 and 34 bacterial and archaeal phyla in the Lō’ihi and EPR datasets, respectively (**Figure 2B**) The community diversity and richness was estimated to be comparable between the two seafloor basalt communities, while being considerably larger than those of subsurface sediment samples (Chao1: ∼63–188; Shannon: 3.3–3.8 at genus-level; Biddle et al., 2011), and approaching those of soil samples (Chao1: 1,400; Shannon: 1.3–4.0; Pearce et al., 2012) (**Figure 2B** – table inset). The microbial community represented by contigs with taxa assignment was dominated by *Gamma-* and *Alphaproteobacteria*, and *Thaumarchaeota* (together 68.6 and 49.6% of total bp at Lō’ihi/EPR, respectively). The *Gammaproteobacterial* class was mostly composed of the ubiquitous marine order *Alteromonadales* (6.6%, 1.0% of total bp at Lō’ihi/EPR), which was previously shown to be among the most abundant orders on seafloor basalts (Jacobson Meyers et al., 2014). *Alphaproteobacteria* was mainly comprised of *Rhizobiales* (0.6 and 0.2% at Lō’ihi/EPR), *Rhodospirillales* (0.2 and 0.02% at Lō’ihi/EPR), *Rhodobacterales* (0.1 and 0.2% at Lō’ihi/EPR), and *Sphingomonadales* (0.2 and 0.1% at Lō’ihi/EPR), which include known seafloor basalt occupying genera (Mason et al., 2007; Jacobson Meyers et al., 2014). The relative abundance of *Thaumarchaeota* sequence assigned by BLAST (12.3 and 26.8% at Lō’ihi/EPR) as well as molecular marker genes (12.8 and 16.0% at Lō’ihi/EPR) was relatively high compared to previous PCR-based seafloor basalt community analyses. Quantitative analyses of microbial communities on the surface of and inside seafloor basalts have reported a ubiquitous but minor presence of archaea, although estimates of archaeal abundance are disparate, ranging from <0.02% at Knipovich Ridge (Einen et al., 2008), 4–12% at Hawai’i, the EPR and the Juan de Fuca Ridge (JdF; Santelli et al., 2008) to 10.6–25.3% at the EPR and the JdF (Mason et al., 2009).

**FIGURE 3:**
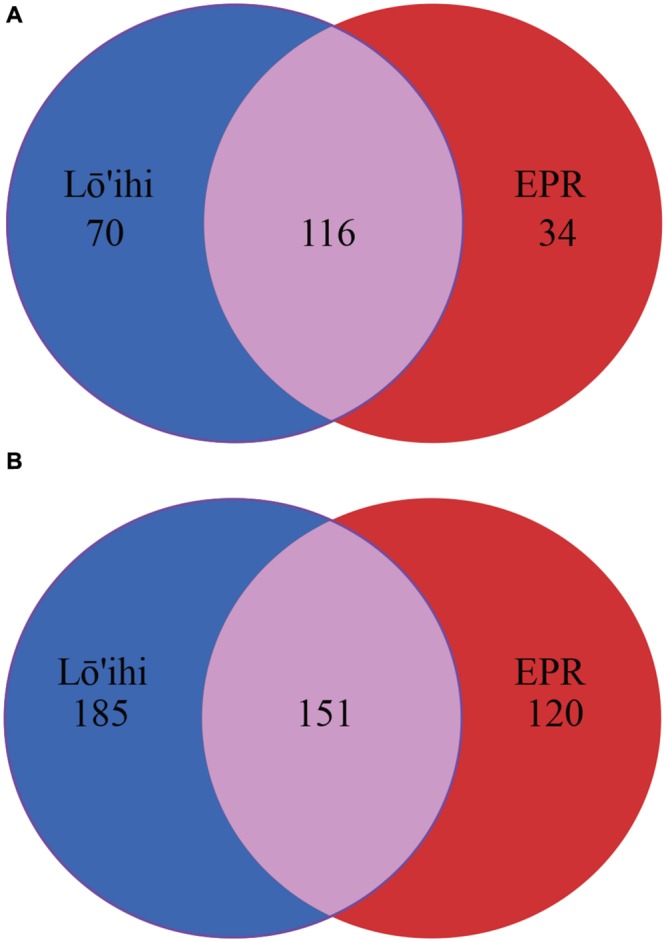
**Venn diagram at family **(A)** and genus **(B)** level.** Numbers represent individual taxonomic groups at their respective phylogenetic rank. Unclassified sequences are excluded from this analysis.

At the EPR, 9.0% of classified contigs were assigned to the *Planctomycetes*, which thereby represent the fourth most abundant phylum (**Figure 2B**). The contigs assigned to the *Planctomycetes* only account for ∼1.0% in the Lō’ihi dataset. Other less represented phyla and *Proteobacterial* classes (1–5%) at both sites include *Betaproteobacteria* (4.0 and 3.4% at Lō’ihi/EPR), *Bacteroidetes* (2.0 and 4.4% at Lō’ihi/EPR), *Delta-/Epsilonproteobacteria* (2.0 and 4.0% at Lō’ihi/EPR), *Firmicutes* (0.9 and 1.5% at Lō’ihi/EPR), *Cyanobacteria* (0.8 and 1.4% at Lō’ihi/EPR), *Verrucomicrobia* (0.6 and 1.1% at Lō’ihi/EPR), as well as *Nitrospirae* (0.1 and 1.5% at Lō’ihi/EPR; Supplementary Table S1). Several phyla were only present at one site: 15 phyla accounting for 0.5% of the total community were only found at the Lō’ihi site, while four phyla accounting for 0.1% were exclusively present at the EPR site. The contigs representing the ‘rare biosphere’ (<1% of total community) are more diverse at Lō’ihi and incorporate 35 phyla (total of 5.1% of total community; Supplementary Table S1). The rare biosphere at the EPR includes 24 phyla representing 4.0% of the total community. At the family level, 18.6 and 27.1% of all Lō’ihi and EPR contigs were classifiable and >50% of all family OTUs overlapped between the two metagenome datasets. At the genus level, 11.8 and 16.5% of all Lō’ihi and EPR contigs were classifiable and >30% of all genus OTUs overlapped between Lō’ihi and EPR (**Figure 3**).

### Functional Genes

The diversity of the microbial communities observed on these seafloor basalts suggests the possibility of the co-occurrence of various nutrient cycles. Functions discussed here were annotated using KEGG Orthology (KO; Supplementary Table S2) and shows that broad categories did not significantly differ in relative abundance between the seafloor basalt metagenome datasets (Supplementary Table S3). In order to determine potential microbial metabolic activities occurring on basalts leading to alteration reactions, we were interested in the type of carbon and energy metabolisms that are predicted by our basalt metagenomes. Since our datasets only represent a fraction of the complete metagenome at Lō’ihi and the EPR, we focused our analysis on the pathways that are present and will rely on future, more comprehensive, gene and pathway studies to resolve ecologically important metabolic functions absent from our dataset.

The transformation potential for organic matter on seafloor basalts was previously estimated to be comparable to that of continental shelf sediments and larger than that in the water column (Jacobson Meyers et al., 2014). Key enzymes involved in carbon fixation, e.g., the large and small subunits of Ribulose-1,5-biosphosphate carboxylase oxygenase (RuBisCo) Form I and II, are assumed to play a dominant role in the accumulation of biomass on basalt surfaces, because carbon concentrations are typically low there (Edwards et al., 2003a). Both, the Lō’ihi and the EPR seafloor basalt metagenomes harbor genes mediating the Calvin-Benson-Bassham (CBB) cycle for autotrophic carbon assimilation, though the ribulose 1,5-biphosphate carboxylase/oxygenase (RuBisCo) gene (*cbbM*) is only present in the Lō’ihi dataset (Supplementary Table S4). The corresponding gene sequence encodes for RuBisCo Form II, which functions at [CO_2_] > 1.5%, and shares closest similarity to that from *Candidatus Ruthia magnifica*, a sulfur-oxidizing endosymbiont retrieved from the deep-sea hydrothermal vent clam *Calyptogena magnifica* (Newton et al., 2007). We also identified a citrate lyase (beta subunit) in the Lō’ihi dataset, which enables the reverse tricarboxylic acid (TCA) cycle. The presence of these genes supports an autotrophic lifestyle on basalts at Lō’ihi. Autotrophy was recently experimentally demonstrated to occur on seafloor basalts from the Lō’ihi Seamount, as well as from North Pond and the Juan de Fuca Ridge via ^13^C incubation experiments (Orcutt et al., 2015). Carbon fixation rates across global oceanic crust were estimated to be 10^9^–10^12^ g C yr^-1^, thereby likely supporting heterotrophic organisms (Orcutt et al., 2015). Genes involved in heterotrophic carbon metabolism are present in both datasets and include chitinases, which degrade chitin, starch phosphorylases, which catalyze phosphorolytic degradation of starch, and beta-1,4-endoglucanases, which hydrolyze cellulose (Supplementary Table S4). Since some of these genes share highest similarity with organisms that are also expected to perform carbon fixation, e.g., starch phosphorylase in *Nitrosomonas cryotolerans*, mixotrophy appears to be a possible alternative lifestyle on seafloor basalts.

**FIGURE 4:**
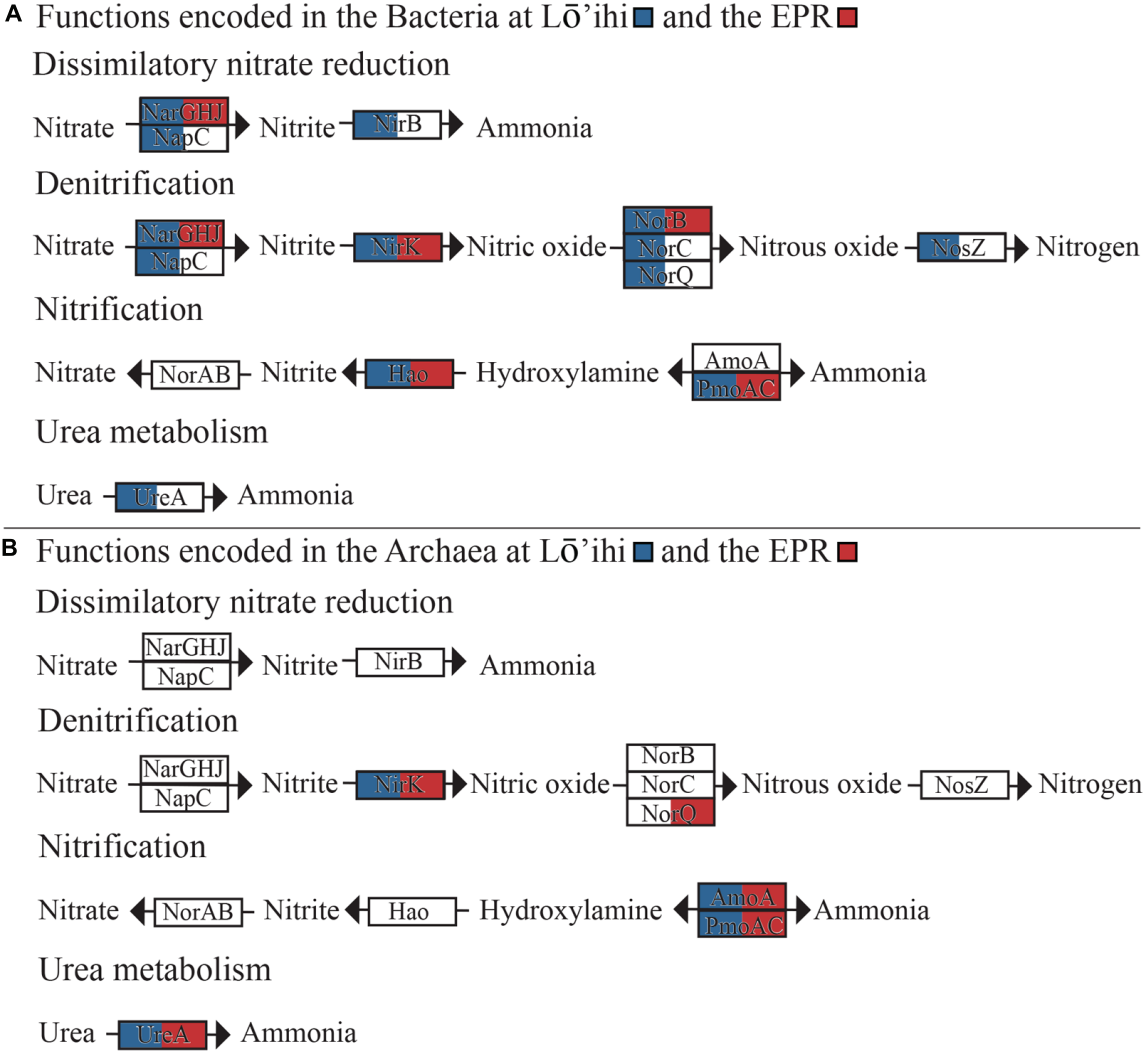
**Nitrogen metabolism pathways encoded in the Lō’ihi and EPR metagenomes attributed to bacteria **(A)** and archaea **(B)**.** Genes present in either metagenome dataset are indicated by coloration of the respective gene boxes (Lō’ihi – blue; EPR – red).

In both datasets, we found bacterial and archaeal genes encoding for various aerobic and anaerobic steps in the nitrogen cycling pathways (**Figure 4**, Supplementary Table S4). These include the complete pathways for dissimilatory nitrate reduction to ammonia (*narGHJ*/*nirB*) and denitrification of nitrate to nitrogen gas (*nirK, norBC, nosZ*). Organisms predicted to be involved in these pathways span *Alpha-, Beta-*, and *Gammaproteobacteria*, as well as *Nitrospirae*, and *Thaumarchaeota* (Supplementary Table S4). The archaeal gene *nirK* shows closest sequence similarity to *nirK* from *Candidatus Nitrosoarchaeum limnia* SFB1 (Lō’ihi) and *Candidatus Nitrosopumilus koreensis* (EPR), therefore likely representing the AnirKa variant (Lund et al., 2012). This variant was previously shown to be more common in the epipelagic to mesopelagic water horizons, but the presence of *nirK* in our deep-sea datasets is an indication of AnirKa in the bathypelagic.

Nitrification is encoded by ammonia monooxygenase subunit C (*amoAC*), hydroxylamine oxidase (*hao*), and nitric oxide reductase (*norAB*). The oxidation of ammonia to hydroxylamine is encoded by *amoC* genes with closest similarity to *Nitrosopumilus sp.* SJ and *Nitrosospira multiformis* in the Lō’ihi as well as the EPR dataset, indicating that both *Thaumarchaeota* and *Betaproteobacteria* may be involved in ammonia oxidation. Pfam motif search also resulted in indications of fractions of *amoA* genes with closest similarity to *amoA* from *Nitrosospira lacus* (56% coverage, 91% identity) and an *amoA* gene from *Thaumarchaeota archaeon* MY2 (78% cov., 96% id.). These findings further support the co-existence of bacterial and archaeal ammonia oxidizing organisms (AOB and AOA) on both host seafloor basalts. Most archaeal contigs with genes involved in nitrogen cycling returned best BLAST hits to members of *Nitrosopumilus* and *Nitrosoarchaeum*, two ubiquitous phyla in the open ocean and coastal waters (Hatzenpichler, 2012), which may significantly contribute to the nitrification process in the deep ocean. Contigs assigned completely to the *Thaumarchaeota* did not harbor any other genes known to facilitate energy metabolism pathways besides those for ammonia oxidation. While genes encoding nitrite reduction and nitrogen fixation to ammonia were not detected in either dataset, we found urease genes with high sequence similarity to urease alpha subunit (*ureA*) genes from bacteria (*Nitrospina, Pseudomonas, Spiribacter*) and archaea (*Nitrosopumilus, Nitrososphaera*). Urea may represent an alternative ammonia source. For example, the soil AOA *Nitrososphaera viennensis* is capable of growth on urea (Tourna et al., 2011). Other studies have suggested that the excess ammonia available for nitrification may be derived from nitrogen fixation (Cowen et al., 2003). However, N_2_ fixation genes were not found in our seafloor basalt metagenomes. Nif protein encoding genes were previously found in basalt microbial communities (Mason et al., 2009) and in crustal fluids attributed to nitrogen fixing, non-methanogenic archaea (Mehta et al., 2005) at the Juan de Fuca Ridge. It is thus likely that nitrogen fixation provides ammonia on seafloor basalts, although this cannot be concluded from our datasets.

Sulfur cycling is a process known to occur in porous basalts due to biological activity (Rouxel et al., 2008). Various gene fractions of *dsrC* and the *dsrEFH* complex were identified with closest similarity to members of the *Proteobacteria*, known to participate in sulfur oxidation, e.g., *Marichromatium purpuratum* (Prange et al., 1999) (Supplementary Table S4). While all sulfate-reducing organisms with *dsrAB* [catalyzing the reduction of sulfite to sulfide (Wagner et al., 1998)], also possess *dsrC*, which interacts with *dsrAB*, DsrC is also a homolog to TusE, a sulfur transfer protein, which can act in a sulfur relay system assumed to be important during sulfur oxidation (Cort et al., 2008). Indeed, DsrC was shown to interact with DsrEFH, and *dsrEFH* genes are specific to sulfur-oxidizing bacteria containing *dsrAB* and do not occur in sulfate-reducers (Cort et al., 2008). Since we did not find any *dsrAB* genes or motifs in either seafloor basalt metagenome, and the gene motif search results in ambiguous annotations, we can only speculate about the simultaneous presence of sulfur oxidation and dissimilatory sulfate reduction reactions on seafloor basalts as previously identified using GeoChip (Mason et al., 2009). As basalts have low concentrations of sulfate, microbial sulfate reduction typically utilizes sulfate from the surrounding seawater. Sulfate reduction and a potential influx of hydrothermal fluids from the underlying crust introduces sulfide into this habitat, which can then abiotically react with ferrous iron to form pyrite (FeS_2_) at low temperatures (Berner, 1964). Although pyrite (FeS_2_) was not detected in basalts from either Lō’ihi or EPR, sulfur oxidation genes, including *dsrEFH* (e.g., from *M. purpuratum* and *Sulfuricella denitrificans*) and molecular marker genes from sulfur-oxidizing as well as sulfate-reducing organisms (e.g., *Sulfitobacter* sp. Bio 11, *Geobacter sulfurreducens, Desulfomonile tiedjei, Thermodesulfobium narugense*) were detected at both sites, supporting the potential for biological sulfate reduction and sulfide oxidation.

Chemolithoautotrophic microbial biomass production in marine basalts has furthermore been attributed to methanogenesis and Fe(II) oxidation (Bach and Edwards, 2003). Genes involved in methanogenesis or methanotrophy were not detected, but we found several species based on molecular marker gene search that are known for methane production under various conditions. These include *Methanocaldococcus* sp., (Jeanthon et al., 1998, 1999; Bellack et al., 2011), *Methanococcus voltae* (Whitman et al., 1982), *Methanothermococcus thermolithotrophicus* (Huber et al., 1982). Species predicted to be capable of methylotrophic growth found in our seafloor basalt datasets include *Methylotenera versatilis* (Kalyuzhnaya et al., 2012), and *Methylophaga* sp., (Kim et al., 2007; Villeneuve et al., 2013). Similarly, although none of the recently identified candidate genes in neutrophilic Fe(II)-oxidation (Barco et al., 2015) were detected in our dataset, a few gene sequences on ambiguously classified contigs showed closest relatedness to sequences from various Z*etaproteobacteria* sp. (0.3%, 0.02% at Lō’ihi/EPR), a group of marine organisms present and often dominant in Fe-rich ecosystems and speculated to contribute a large amount of Fe(III)-oxides to the global oceans (Singer et al., 2011; Barco et al., 2015).

## Discussion

Ecology studies of seafloor basalts attempting to link phylogenetic players to metabolic function are scarce to date. Seafloor basalts have previously been recognized to host a microbial phylogenetic diversity that is richer than the typical surrounding seawater and to show considerable taxonomic overlap between basalt sites of similar age and alteration state (Santelli et al., 2008). It seems that the geographical location of seafloor basalts only initially influences microbial community composition, and that mineralogy is the driving force that determines how a community structure develops and catalyzes the weathering of its host rock (Toner et al., 2013). Seafloor basalts from both sites discussed here were formed at approximately the same time (∼20,000 years ago) and were selected to confirm the hypothesis of community similarity among like basalts. XRD analysis confirmed an average mineralogical composition, with Lō’ihi being enriched in olivine and pyroxene, and generally containing higher Fe(II) compared to the EPR. Phylogenetic analysis demonstrated considerable overlap between Lō’ihi and EPR on various phylogenetic levels and even showed matches to many of the same dominant species, including members of the *Thaumarchaeota*. The large relative abundance of archaea detected in our datasets is comparable between sites and slightly higher compared to those of previous (PCR-dependent) studies. In fact, in the EPR dataset, *Thaumarchaeota* represented the second most abundant phylum with 12–27% of total sequence volume.

Predicted metabolic functions display comparable diversity and suggest that lithoautotrophic and heterotrophic lifestyles occur next to each other on the host seafloor basalts. The potential for exposed basalts to transform organic matter was previously shown to be substantial, confirming this environment is a major player in benthic biogeochemical processes (Jacobson Meyers et al., 2014; Orcutt et al., 2015). Basalt mineralogy is not likely to fuel all of the encoded metabolic activities, but may support, for instance, sulfate-reducing organisms during the interaction with seawater in reducing micro-niches on and inside the seafloor basalt. The genetic potential for the co-existence of aerobic and anaerobic metabolic processes, e.g., denitrification and nitrification, has been observed in various marine environments, such as oil seeps (Hawley et al., 2014), oxygen minimum zones (Yan et al., 2012; Kalvelage et al., 2013), and deep-sea sedimentary environments (Nunoura et al., 2013). Nitrogen cycling, especially the aerobic oxidation of ammonia, is more commonly encoded in the archaeal fraction of the metagenomes compared to the other studied processes and shows the potential for competition between bacteria and archaea for ammonia. The relative importance of AOA in nitrification as compared to AOB has been debated in other environments (Hatzenpichler, 2012), and similarly requires more quantitative data for the seafloor basalt environment. Interestingly, there were no entire contigs that could be unambiguously assigned to the *Zetaproteobacteria*, and BLAST searches of known genes involved in Fe(II)-oxidation at circumneutral pH did not return any hits in our dataset. While previous studies identified *Zetaproteobacteria* as abundant, and sometimes dominant Fe(II)-oxidizing members of the seafloor basalt community, e.g., (McAllister et al., 2011), *Zetaproteobacteria* sequences may not have been assembled into contigs in our datasets because of low relative abundance, high diversity of *Zetaproteobacteria* genomic information and/or MDA bias. Testing the latter would be useful for future low biomass metagenomic studies on seafloor basalts that are assumed to host a significant abundance of *Zetaproteobacteria.*

This study is a first attempt at characterizing the seafloor basalt environment using a metagenome approach. The insights gained here contribute to addressing the question of ‘who is doing what?’, which remains a challenge, as many sequences are novel compared to what is in our current databases and are consequently assembled or functionally placed with low confidence. It appears that a broad range of bacterial phyla gives rise to a similarly broad range of metabolic gene potentials, whereas archaeal functional genes were mainly found to encode ammonia and methane oxidation. Our qualitative and quantitative interpretation of the results is certainly subject to the incompleteness of our datasets as well as the bias associated with the MDA process (Yilmaz et al., 2010). Hence, further examination of organisms responsible for seafloor basalt alteration and general redox reactions can be achieved in more detail with more in-depth sequencing and upon the availability of more genomes that are environmentally significant in the seafloor basalt habitat.

## Conflict of Interest Statement

The authors declare that the research was conducted in the absence of any commercial or financial relationships that could be construed as a potential conflict of interest.

## Supplementary Material

Figure S1 **Contig length by sample. (A)** Lō’ihi **(B)** EPR **(C)** NC.
