# Supplementary material for: Similar Microbial Communities Found on Two Distant Seafloor Basalts
Source: Front Microbiol. 2015 Dec 16;6:1409. doi: 10.3389/fmicb.2015.01409 (PMC4679871; doi:10.3389/fmicb.2015.01409)
Supplement: Supplementary file 1 [file Table_1.DOCX]

Supplementary Table S1: Complete list of phyla and relative sequence abundances at the Lō’ihi Seamount and the EPR. Phyla exclusively found in one dataset are marked in bold.

| **Phylum** | **Lō’ihi** | **EPR** |
| --- | --- | --- |
| Gammaproteobacteria | 32.24% | 10.27% |
| Alphaproteobacteria | 24.04% | 12.56% |
| Thaumarchaeota | 12.27% | 26.81% |
| Not assigned | 11.32% | 14.37% |
| Viruses | 4.82% | 3.87% |
| Betaproteobacteria | 3.97% | 3.42% |
| Delta/epsilon subdivisions | 1.97% | 4.01% |
| Bacteroidetes | 1.95% | 4.38% |
| Unclassified Bacteria | 1.30% | 1.97% |
| Planctomycetes | 1.01% | 9.00% |
| Firmicutes | 0.90% | 1.47% |
| Cyanobacteria | 0.83% | 1.42% |
| Verrucomicrobia | 0.60% | 1.10% |
| Actinobacteria | 0.37% | 0.85% |
| **Chlamydiae** | 0.34% | 0.00% |
| Zetaproteobacteria | 0.31% | 0.02% |
| Unclassified Proteobacteria | 0.30% | 0.19% |
| Acidobacteria | 0.26% | 0.46% |
| **Hydrogenedentes** | 0.21% | 0.00% |
| Nitrospinae | 0.20% | 0.45% |
| Chloroflexi | 0.16% | 0.44% |
| Nitrospirae | 0.09% | 1.32% |
| **Chlorobi** | 0.06% | 0.00% |
| Euryarchaeota | 0.05% | 0.33% |
| Unclassified Archaea | 0.04% | 0.30% |
| Thermotogae | 0.04% | 0.04% |
| **Deferribacteres** | 0.04% | 0.00% |
| **Thermodesulfobacteria** | 0.03% | 0.00% |
| **Gracilibacteria** | 0.03% | 0.00% |
| Deinococcus-Thermus | 0.02% | 0.08% |
| **Latescibacteria** | 0.02% | 0.00% |
| Aquificae | 0.02% | 0.13% |
| **Poribacteria** | 0.02% | 0.00% |
| Spirochaetes | 0.02% | 0.19% |
| Crenarchaeota | 0.02% | 0.04% |
| Marinimicrobia | 0.02% | 0.04% |
| Caldiserica | 0.02% | 0.16% |
| **Elusimicrobia** | 0.02% | 0.00% |
| Atribacteria | 0.01% | 0.03% |
| **Chrysiogenetes** | 0.01% | 0.00% |
| **Diapherotrites** | 0.01% | 0.00% |
| **Fervidibacteria** | 0.01% | 0.00% |
| **Microgenomates** | 0.01% | 0.00% |
| Caldithrix | 0.01% | 0.02% |
| Lentisphaerae | 0.01% | 0.13% |
| **Ignavibacteriae** | 0.01% | 0.00% |
| **Candidate** **division** **NC10** | 0.01% | 0.00% |
| **Gemmatimonadetes** | 0.00% | 0.03% |
| **Candidate division BRC1** | 0.00% | 0.02% |
| **Nanohaloarchaeota** | 0.00% | 0.02% |
| **Aminicenantes** | 0.00% | 0.05% |
