# Supplementary material for: Similar Microbial Communities Found on Two Distant Seafloor Basalts
Source: Front Microbiol. 2015 Dec 16;6:1409. doi: 10.3389/fmicb.2015.01409 (PMC4679871; doi:10.3389/fmicb.2015.01409)
Supplement: Supplementary file 2 [file Table_2.DOCX]

Supplementary Table S2: KO pathway abundances based on BLAST identified hits per sample

| **KEGG pathway** | **Lō’ihi** | **EPR** | **Blank** |
| --- | --- | --- | --- |
| Carbohydrate metabolism | 11.9% | 13.2% | 12.2% |
| Energy metabolism | 9.5% | 10.9% | 4.3% |
| *Oxidative phosphorylation* | 2.4% | 3.1% | 2.1% |
| *Photosynthesis* | 0.1% | 0.1% | 0.5% |
| *Carbon fixation in photosynthetic organisms* | 0.7% | 0.7% | 0.4% |
| *Carbon fixation pathways in prokaryotes* | 1.6% | 1.8% | 0.9% |
| *Methane metabolism* | 1.4% | 1.9% | 1.0% |
| *Nitrogen metabolism* | 2.9% | 3.0% | 1.2% |
| *Sulfur metabolism* | 0.3% | 0.4% | 0.4% |
| Lipid metabolism | 2.6% | 2.8% | 2.1% |
| Nucleotide metabolism | 4.9% | 4.7% | 3.6% |
| Amino acid metabolism | 12.3% | 13.5% | 8.3% |
| Metabolism of other amino acids | 2.3% | 2.0% | 2.7% |
| Glycan biosynthesis and metabolism | 1.3% | 1.0% | 1.8% |
| Metabolism of cofactors and vitamins | 5.0% | 4.9% | 1.6% |
| Metabolism of terpenoids and polyketides | 1.5% | 1.4% | 0.5% |
| Biosynthesis of other secondary metabolites | 0.7% | 1.2% | 1.7% |
| Xenobiotics biodegradation and metabolism | 2.7% | 2.2% | 1.6% |
| Genetic information processing | 8.6% | 9.5% | 5.0% |
| *Transcription* | 0.4% | 0.6% | 0.0% |
| *Translation* | 3.1% | 3.3% | 0.9% |
| *Folding, sorting and degradation* | 2.0% | 2.0% | 1.5% |
| *Replication and repair* | 3.1% | 3.6% | 2.7% |
| Environmental information processing | 5.5% | 4.3% | 10.7% |
| *Membrane transport* | 2.3% | 2.6% | 6.9% |
| *Signal transduction* | 3.2% | 1.7% | 3.8% |
| *Signaling molecules and interaction* | 0.0% | 0.0% | 0.0% |
| Cellular processes | 2.5% | 1.0% | 2.6% |
| *Transport and catabolism* | 0.3% | 0.3% | 0.4% |
| *Cell motility* | 1.5% | 0.3% | 2.1% |
| *Cell growth and death* | 0.7% | 0.3% | 0.1% |
| *Cell communication* | 0.0% | 0.0% | 0.0% |
| Organismal systems | 1.4% | 1.3% | 1.5% |
| Human diseases | 2.8% | 2.5% | 2.2% |
| Unclassified | 24.6% | 23.5% | 37.6% |
