# Supplementary material for: Similar Microbial Communities Found on Two Distant Seafloor Basalts
Source: Front Microbiol. 2015 Dec 16;6:1409. doi: 10.3389/fmicb.2015.01409 (PMC4679871; doi:10.3389/fmicb.2015.01409)
Supplement: Supplementary file 3 [file Table_3.DOCX]

Supplementary Table S3: D-score calculation of functional categories on the basis of functional gene abundance in each category. *D*-scores > 1.96 reject the null hypothesis and indicate significant differences (equivalent to a p-value < 0.05). Representation of functional categories is not significantly different between Lō’ihi and the EPR.

| **KEGG pathway** | **Lō’ihi** | **EPR** |
| --- | --- | --- |
| Carbohydrate metabolism | -0.749 | 0.251 |
| Energy metabolism | -0.799 | 0.201 |
| *Oxidative phosphorylation* | -0.390 | 0.610 |
| *Photosynthesis* | 0.019 | 0.981 |
| *Carbon fixation in photosynthetic organisms* | 0.007 | 0.993 |
| *Carbon fixation pathways in prokaryotes* | -0.081 | 0.919 |
| *Methane metabolism* | -0.266 | 0.734 |
| *Nitrogen metabolism* | -0.042 | 0.958 |
| *Sulfur metabolism* | -0.062 | 0.938 |
| Lipid metabolism | -0.159 | 0.841 |
| Nucleotide metabolism | 0.069 | 0.931 |
| Amino acid metabolism | -0.703 | 0.297 |
| Metabolism of other amino acids | 0.178 | 0.822 |
| Glycan biosynthesis and metabolism | 0.192 | 0.808 |
| Metabolism of cofactors and vitamins | 0.044 | 0.956 |
| Metabolism of terpenoids and polyketides | 0.065 | 0.935 |
| Biosynthesis of other secondary metabolites | -0.298 | 0.702 |
| Xenobiotics biodegradation and metabolism | 0.335 | 0.665 |
| Genetic information processing | -0.532 | 0.468 |
| *Transcription* | -0.142 | 0.858 |
| *Translation* | -0.112 | 0.888 |
| *Folding, sorting and degradation* | 0.003 | 0.997 |
| *Replication and repair* | -0.288 | 0.712 |
| Environmental information processing | 0.711 | 0.289 |
| *Membrane transport* | -0.158 | 0.842 |
| *Signal transduction* | 0.881 | 0.119 |
| *Signaling molecules and interaction* | -0.007 | 0.993 |
| Cellular processes | 0.883 | 0.117 |
| *Transport and catabolism* | 0.030 | 0.970 |
| *Cell motility* | 0.671 | 0.329 |
| *Cell growth and death* | 0.192 | 0.808 |
| *Cell communication* | -0.007 | 0.993 |
| Organismal systems | 0.039 | 0.961 |
| Human diseases | 0.125 | 0.875 |
| Unclassified | 0.625 | 0.375 |
