# Supplementary material for: Similar Microbial Communities Found on Two Distant Seafloor Basalts
Source: Front Microbiol. 2015 Dec 16;6:1409. doi: 10.3389/fmicb.2015.01409 (PMC4679871; doi:10.3389/fmicb.2015.01409)
Supplement: Supplementary file 4 [file Table_4.DOCX]

**Supplementary Table S4: Functional gene analysis using KEGG Orthology (KO) and Pfam annotation pipelines.** Listed are taxa details of closest BLAST hits, contig IDs and bit score as well as e-values in round brackets, in order of largest bit score.

| **Function** | **Pathway** | **Gene** | **KO or Pfam id** | **Lō’ihi count** | **Taxon** | | **EPR count** | **phylum** | |
| --- | --- | --- | --- | --- | --- | --- | --- | --- | --- |
| **Carbon metabolism** | | | | | | | | | |
| Ribulose biphosphate carboxylase large chain, catalytic domain | Carbon fixation | *cbbM* | PF00016.15 | 1 | *Sulfuricella denitrificans*  (c1860): 325.6 (3.2E-97) | 0 | | |  |
| Ribulose biphosphate carboxylase large chain, N-terminal domain | Carbon fixation | *cbbM* | PF02788.11 | 1 | *Sulfuricella denitrificans*  (c1860): 111.0 (2.7E-32) | 0 | | |  |
| Citrate lyase | Reverse TCA cycle | *citF* | K01643 | 1 | *Sneathiella glossodoripedis*  (c6802): 312.4 (0.0) | 0 | | |  |
| Chitinase | Amino sugar and nucleotide sugar metabolism |  | K01183 | 1 | *Psedualteromonas* sp. (c12486): 368.2 (0.0) | 1 | | | *Aquimarina megaterium* (c5521): 184.1 (2.0E-43) |
| Starch phosphorylase | Starch and sucrose metabolism | *glgP* | K00688 | 3 | *Nitrosomonas* sp. (c10621): 351.3 (0.0)  *Nitrosomonas* sp. (c11242): 339.0 (0.0)  *Latescibacteria bacterium* (c553): 216.9 (0.0) | 4 | | | *Nitrosomonas cryotolerans* (c1442): 278.1 (0.0)  Candidatus *Nitrospira defluvii* (c2009): 250.8 (0.0)  Candidatus *Nitrospira defluvii* (c6859): 212.2 (0.0)  *Pirellula staleyi* (c4): 139.4 (6.4E-30) |
| Beta-1,4-Endoglucanase | Starch and sucrose metabolism |  | K01179 | 3 | Candidatus *Kuenenia stuttgartiensis* (c16512): 188.0 (1.7E-44)  *Aerophobetes bacterium* (c14551): 156.8 (3.0E-35)  *Geopsychrobacter electrodiphilus* (c10365): 110.9 (2.1E-21) | 3 | | | *Acidovorax citrulli* (c711): 310.1 (0.0)  *Acidovorax citrulli* (c1603): 156.0 (9.9E-35)  *Acidovorax avenae* (c8593): 103.6 (3.3E-19) |
| **Nitrogen metabolism** | | | | | | | | | |
| Nitrate reductase (alpha subunit) | Diss nitrate reduction, denitrification | *narG* | K00370 | 3 | *Marinobacter lipolyticus* (c10761): 298.9 (0.0),  *Methylobacter tundripaludum* (c10816): 253.4 (0.0),  *Neptunomomas japonica* (c16828): 402.9 (0.0) | | 1 | *Arenibacter algicola* (c6409): 178.3 (1.0E-41) | |
| Nitrate reductase (beta subunit) | Diss nitrate reduction, denitrification | *narH* | K00371 | 2 | (*Calyptogena okutanii thioautotrophic gill symbiont*) (c11871): 299.7 (0.0), *Maricaulis maris* ( c2242): 391.0 (0.0) | | 1 | *Ochrobactrum anthropi* (c8094): 273.5 (0.0) | |
| Nitrate reductase (delta subunit) | Diss nitrate reduction, denitrification | *narJ* | K00373 | 2 | *Brevundimonas* sp. (c10700): 112.1 (1.0E-21),  Calyptogena okutanii thioautotrophic gill symbiont (c2781): 203.4 (0.0) | | 0 |  | |
| Nitrite reductase | Diss nitrate reduction | *nirB* | K00362 | 2 | *Thioalkalivibrio* sp. (c622): 320.5 (0.0),  *Thalassospira profundimaris* (c5784): 174.5 (1.6E-40) | |  |  | |
| Cytochrome C nitrite reductase | Diss nitrate reduction | *napC*/*nirT* | PF03264.9 | 5 | *Oleispira antarctica* (c16158): 346 (2E-111),  Uncultured Gammaproteobacterium (c6123): 64.8 (6.1E-18),  *Nitrosospira* sp. (c13226): 114.9 (2.6E-33),  *Sedimenticola selenatireducens* (c11968): 140.2 (4.3E-41),  Acidithiobacillales bacterium (c1631): 223.3 (1.2E-66) | | 1 | *Nitrospira defluvii* (c2550): 305 (8E-100) | |
| Nitrite reductase (NO-forming) | (De-)nitrification | *nirK* | K00368 | 4 | *Nitrosomonas* sp. (c12476): 225.7 (0.0),  Candidatus *Nitrosoarchaeum limnia* (c3927): 354.4 (0.0), *Nitrosopumilus* (c6137): 405.2 (0.0),  *Nitrosomonas cryotolerans* (c7953): 419.1 (0.0) | | 2 | Candidatus *Nitrosopumilus koreensis* (c524): 530.0 (0.0), *Rhodopseudomonas* sp. (c2512): 281.6 (0.0) | |
| Nitric oxide reductase subunit B | Denitrification | *norB* | K04561 | 1 | *Azospirillum lipoferum* (c942): 686.0 (0.0) | | 1 | *Rhodopirellula europaea* (c3689): 460.3 (0.0) | |
| Nitric oxide reductase subunit C | Denitrification | *norC* | K02305 | 1 | *Rhizobium* (c3880): 184.1 (2.8E-43) | | 0 |  | |
| Nitrous-oxide reductase | Denitrification | *nosZ* | K00376 | 2 | *Brucella melitensis* (c2169): 481.5 (0.0),  *Shewanella* sp. (c9216): 301.6 (0.0) | | 0 |  | |
| Nitric oxide reductase | Denitrification | *norQ* | K04748 | 2 | *Beggiatoa alba* (c13990): 147.5 (2.2E-32), *Methylotenera* sp. (c11515): 153.7 (34E-34) | | 1 | *Nitrosopumilus* (c5346): 120.2 (4.0E-24) | |
| Hydroxylamine oxidase | Nitrification | *hao* | K10535 | 2 | Endosymbiont of Tevnia jerichonana (c10398): 340.9 (0.0),  Endosymbiont of Riftia pachyptila (c8621): 238.4 (0.0) | | 1 | *Nitrosomonas cryotolerans* (c4494): 481.1 (0.0) | |
| Ammonia monooxygenase subunit A | Nitrification | *amoA* | PF12942.2 | 2 | Uncultured Crenarchaeote (c5281): 318.0 (2E-106),  Uncultured Crenarchaeote (c8131): 222.0 (1.0E-68) | | 1 | Uncultured Crenarchaeote (c1538): 226 (2.8E-67) | |
| Ammonia monooxygenase subunit C | Nitrification | *amoC* | K10946 | 2 | *Nitrosopumilus* sp. (c17046): 326.6 (0.0),  *Nitrosospira multiformis* (c8983): 495.4 (0.0) | | 3 | *Nitrosospira multiformis* (c298): 310.8 (0.0),  *Nitrosospira multiformis* (c4000): 441.4 (0.0),  Candidatus *Nitrosopumilus* *salaria* (c4192): 298.1 (0.0) | |
| Urease, alpha subunit | Urea metabolism | *ureA* | K01428 | 5 | *Nitrososphaera viennensis* (c2240): 812.8 (0.0),  *Spiribacter salinus* (c5792): 290.0 (0.0),  *Nitrosopumilus* (c8649): 242.7 (0.0),  *Pseudomonas denitrificans* (c4946): 235.3 (0.0),  *Nitrospina* sp. (c15212): 90.9 (2.4e-15), | | 1 | *Nitrosopumilus* sp. (c699): 870.5 (0.0) | |
| Nitrate/nitrite sensor histidine kinase | Nitrate respiration | *narX*/*narL* | K07673 | 3 | *Thioalkalivibrio nitratireducens* (c13655): 141.4 (1.4E-30),  *Thioalkalivibrio sulfidiphilus* (c1277): 298.1 (0.0),  Solemya velum gill symbiont (c1401): 313.9 (0.0) | | 0 |  | |
| Formate dehydrogenase | Formate metabolism |  | K00122, K00123, K00124,  K00127 |  | *Ensifer sojae* (c16276): 348.2 (0.0),  *Burkholderia* sp. (c15615): 250.4 (0.0),  *Desulfospira joergensenii* (c3682): 184.9 (1.79E-43),  *Geminicoccus roseus* (c7787): 107.1 (2.6E-20),  *Azospirillum* sp. (c6292): 370.5 (0.0),  *Herbaspirillum* sp. (c9560): 162.2 (1.0E-36) | | 2 | *Silicibacter* sp. (c6867): 276.2 (0.0)  *Alkalilimnicola ehrlichii* (c8778): 198.0 (0.0) | |
| **Sulfur metabolism** | | | | | | | | | |
| DsrC like protein, TusE | Sulfur oxidation, sulfur transfer protein | *dsrC*/*tusE* | PF04358.8 | 3 | *Sedimenticola selenatireducens* (c1512): 113.3 (6.0E-33),  *Sulfuricella denitrificans*  (c16720): 99.1 (1.6E-28) | | 1 | *Marichromatium purpuratum* (c6200): 110.9 (3.3E-32) | |
| DsrE/DsrF/DrsH-like family | Sulfur oxidation | *dsrEFH* | PF02635.10,  PF13686.1 | 4 | *Thiohalorhabdus denitrificans*  (c988): 64.6 (7.9E-18),  Uncultured Gammaproteobacterium (c7554): 75 (4.8E-21),  Uncultured bacterium (c9784): 76.2 (2.1E-21),  Uncultured Deltaproteobacterium (c11628): 94.2 (6.0E-27) | | 1 | *Marichromatium purpuratum* (c6200): 66.9 (8.7E-19) | |
| Sulfur oxidation protein | Thiosulfate oxidation by SOX complex | *soxY* | PF13501.1 | 1 | *Sedimenticola* sp. (c6042): 131.3 (1.4E-38) | | 0 |  | |
| Methane cycling |  |  |  |  |  | |  |  | |
| Methane oxygenase |  | *pmoA* | PF14100.1 |  |  | | 1 | *Rhodopirellula* sp. (c8675): 75.6 (3.5E-21) | |
| Methane monooxygenaes |  | *pMMO* | PF02461.11 |  |  | | 1 | *Nitrosospira lacus* (c4935): 262 (3E-83) | |
