# Supplementary figures and images for: Similar Microbial Communities Found on Two Distant Seafloor Basalts
Source: Front Microbiol. 2015 Dec 16;6:1409. doi: 10.3389/fmicb.2015.01409 (PMC4679871; doi:10.3389/fmicb.2015.01409)

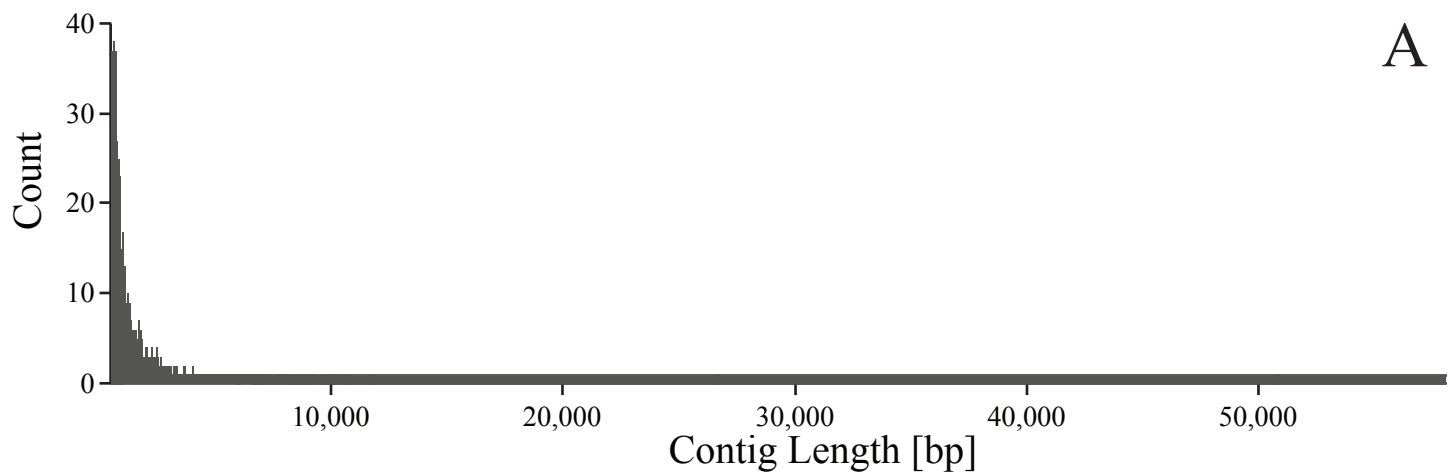

Supplement: Figure S1 — Contig length by sample. (A) Lō’ihi (B) EPR (C) NC. [file Image_1.PDF]
